# Triglycerides/HDL cholesterol ratio and type 2 diabetes incidence: Panasonic Cohort Study 10

**DOI:** 10.1186/s12933-023-02046-5

**Published:** 2023-11-08

**Authors:** Hiroki Yuge, Hiroshi Okada, Masahide Hamaguchi, Kazushiro Kurogi, Hiroaki Murata, Masato Ito, Michiaki Fukui

**Affiliations:** 1 Department of Endocrinology and Metabolism, Graduate School of Medical Science, Kyoto Prefectural University of Medicine, 465 Kajii-Cho, Kawaramachi-Hirokoji, Kamigyo-Ku, Kyoto, 602-8566 Japan; 2 Department of Diabetes and Endocrinology, Matsushita Memorial Hospital, 5-55 Sotojima-Cho, Moriguchi, 570-8540 Japan; 3 Department of Health Care Center, Panasonic Health Insurance Organization, 5-55 Sotojima-Cho, Moriguchi, 570-8540 Japan; 4 Department of Orthopedic Surgery, Matsushita Memorial Hospital, 5-55 Sotojima-Cho, Moriguchi, 570-8540 Japan

## Abstract

**Background:**

Previous studies have investigated the association between the ratio of triglycerides (TG) to high-density lipoprotein cholesterol (HDL-C) and the incidence of diabetes in adults and discovered that a high TG/HDL-C ratio was linked to an elevated risk of new-onset diabetes. However, the comparison of predicting diabetes development among lipid profiles including the TG/HDL-C ratio, and the ratio of TG/HDL-C cut-off value has received limited attention. We examined the relationship between diabetes onset and the TG/HDL-C ratio in addition to the applicable cut-off value for predicting diabetes onset.

**Methods:**

This study included 120,613 participants from the health examination database at Panasonic Corporation from 2008 to 2017. Cox regression analysis employing multivariable models was used to investigate the association between lipid profiles, particularly the ratio of TG/HDL-C and the development of type 2 diabetes (T2D). The multivariable model was adjusted for age, sex, BMI, systolic blood pressure, plasma glucose levels after fasting, smoking status, and exercise habits. Areas under time-dependent receiver operating characteristic (ROC) curves (AUCs) were employed to assess the prediction performance and cut-off values of each indicator. A fasting plasma glucose level of 126 mg/dL, a self-reported history of diabetes, or usage of antidiabetic medicines were used to identify T2D.

**Results:**

During the course of the study, 6,080 people developed T2D. The median follow-up duration was 6.0 (3–10) years. Multivariable analysis revealed that the ratio of TG/HDL-C (per unit, HR; 1.03 [95% CI 1.02–1.03]) was substantially linked to the risk of incident T2D. AUC and cut-off points for the ratio of TG/HDL-C for T2D development after 10 years were 0.679 and 2.1, respectively. Furthermore, the AUC of the ratio of TG/HDL-C was considerably larger compared to that of LDL-C, HDL-C, and TG alone (all P < 0.001). We discovered an interaction effect between sex, BMI, and lipid profiles in subgroup analysis. Females and participants having a BMI of < 25 kg/m^2^ showed a higher correlation between lipid profile levels and T2D onset.

**Conclusions:**

The ratio of TG/HDL-C was found to be a stronger predictor of T2D development within 10 years than LDL-C, HDL-C, or TG, indicating that it may be useful in future medical treatment support.

**Supplementary Information:**

The online version contains supplementary material available at 10.1186/s12933-023-02046-5.

## Background

Diabetes prevalence among individuals aged 20–79 was predicted to be 10.5% (536.6 million) in 2021, escalating to 12.2% (783.2 million) in 2045 worldwide [1]. Diabetes is a risk factor for atherosclerosis and has been related to an increased risk of death from several cancers, infectious diseases, and cardiovascular diseases [2]. Diabetes-related healthcare costs worldwide are expected to reach US$ 966 billion in 2021 and US$ 1,054 billion by 2045 [1]. Thus, the development of diabetes and related complications, as well as the associated increase in healthcare expenses, are worldwide issues, making it critical to manage the onset of diabetes to avoid progression of complications.

Lipid metabolic abnormalities, on the other hand, have been related to an increased likelihood of developing cardiovascular disease [3,4]. Diabetes dyslipidemia is defined by high triglyceride (TG) levels, low high-density lipoprotein cholesterol (HDL-C) levels, and high low-density lipoprotein cholesterol (LDL-C) values, especially small dense LDL-C. Moreover, although abnormalities in lipid metabolism affect diabetes development [5,6], the evidence regarding the association between dyslipidemia and type 2 diabetes (T2D), including cut-off values, is still lacking.

Previous research has revealed that the ratio of TG/HDL-C is an accurate predictor of insulin resistance [7–9]. The ratio of TG/HDL-C has also been researched in relation to other events; it raises the chance of developing fatty liver that is independent of other factors [10], and correlates positively with arterial stiffness [11,12]. Previous studies have investigated the association between the ratio of TG/HDL-C and the incidence of diabetes in adults, and discovered that a higher ratio of TG/HDL-C was linked to an elevated risk of new-onset diabetes [13–18]. Zhou et al. [19] reported the cut-off values of TG/HDL-C ratio for predicting the development of T2D; however, theirs was a cross-sectional study, leaving the causal relationship unclear. One study reported the cut-off values of the TG/HDL-C ratio for predicting the incidence of T2D [18], but the sample size was small, the observation period was not taken into account, and the performance among lipid profiles for predicting T2D incidence was not compared [18]. To the best of our knowledge, the comparison of predicting diabetes development among lipid profiles, including the ratio of TG/HDL-C and the ratio of TG/HDL-C cut-off value, has received little attention.

Consequently, we undertook this cohort study conducted in the past to investigate the relationship between the lipid profile, including the ratio of TG/HDL-C, alongside the development of diabetes over 10 years, and to estimate the ratio of TG/HDL-C cut-off value for predicting T2D development.

## Methods

### Study population and study design

This long-term retrospective cohort study used data from the Panasonic Cohort Study, which were collected between 2008 and 2018. This database contains information on annual medical health checks, medical costs, medical history, and mortality among Panasonic Corporation employees in Osaka, Japan. This examination program was designed to promote the health of employees by detecting chronic diseases early, such as metabolic abnormalities, and evaluating the underlying risk factors. All employees received yearly medical health checks.

Blood samples were taken following > 10 h of fasting. A weight and height meter that is automatic was used to record the subjects' weight and height. A previously established and verified self-administered questionnaire was employed to assess baseline features, including smoking habits, exercise habits, history of medical treatment, and history of diabetes. Three groups of participants were formed: nonsmokers, former smokers, and current smokers. Regular exercisers were those who engaged in at least 30 min of exercise at least 2 days per week for at least a year. A fasting plasma glucose level of 126 mg/dL, a self-reported history of diabetes, or usage of antidiabetic medicines were used to identify T2D.

This study was approved by the Panasonic Health Insurance Organization's local ethics committee (permission number: 2021–001) and was carried out in conformity with the principles of the Helsinki Declaration.

### Inclusion and exclusion criteria

**Fig. 1:**
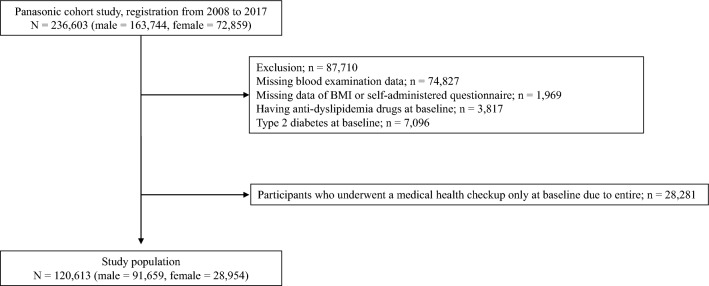
Study flow diagram for the registration of participants

Figure 1 depicts the participant registration flow diagram for the study. Employees who had medical health checks between 2008 and 2017 were all registered. Between 2008 and 2017, a total of 236,603 employees had medical health checks. The observation period for the development of T2D was set until 2018. Participants who did not have a blood test at baseline, those with incomplete data, those taking antidyslipidemic medicines at baseline, those with diabetes at baseline, and those who had undergone a medical health checkup at baseline only because they were retiring were excluded from the study.

### Statistical analyses

The potential confounding variables' means and frequencies were computed. All potential confounding variables were baseline values. The primary outcome measure was the relationship between lipid profiles, especially the ratio of TG/HDL-C, and T2D incidence. The T2D incidence and lipid profiles were evaluated in the annual medical health checks. Cox regression analysis with multivariable models was utilized to investigate the connection between LDL-C, HDL-C, TG, and the ratio of TG/HDL-C and T2D incidence. The multivariable model was adjusted for age, sex, BMI, systolic blood pressure, plasma glucose levels after fasting, smoking status, and exercise habits. We also conducted a likelihood ratio test to examine whether lipid profiles have a significant prognostic impact. We used time-dependent ROC curves for censored survival data and AUCs as criteria to assess the predictive performance of LDL-C, HDL-C, TG, and the ratio of TG/HDL-C. The best cut-off values for LDL-C, HDL-C, TG, and the ratio of TG/HDL-C for incident T2D were investigated. Moreover, we compared the AUCs of LDL-C, HDL-C, TG, and ratios of TG/HDL-C using 1,000 bootstrap samples with the Bonferroni method.

The impact of sex and BMI was evaluated using a subgroup analysis. The subgroups were categorized as male and female, and individuals with BMI < 25 kg/m^2^ and ≥ 25 kg/m^2^, according to the Japan Society for the Study of Obesity's definition. Except for sex or BMI, the multivariable model was adjusted for variables in subgroup analyses as appropriate. Hazard ratios (HRs), AUCs, and cut-off values were evaluated according to sex and BMI category. We also tested the potential interaction effects of sex, BMI category, and lipid profile.

The mean, standard deviation, or absolute numbers are used to represent all continuous variables. At *P* < 0.05, differences were judged to be statistically significant. When the Bonferroni method was applied, differences were considered statistically significant at P < 0.008. HRs with 95% confidence intervals (CIs) were used to represent associations. JMP software version 17 (SAS Institute, Cary, NC, USA) was used for statistical analyses.

## Results

### Main analyses

**Fig. 2:**
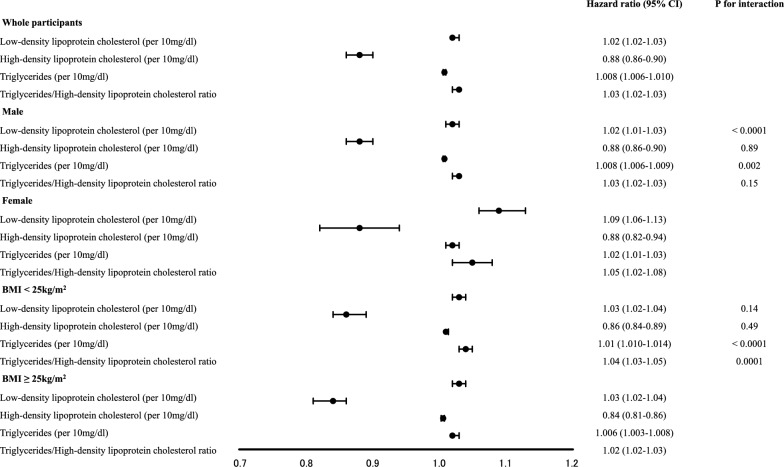
Adjusted hazard ratios for incidence of diabetes during the 10-year follow-up period in whole participants, male and female, and participants with BMI < 25 kg/m^2^ and BMI ≥ 25 kg/m^2^

The analysis included 120,613 participants in total (Fig. 1). The baseline characteristics of the individuals are depicted in Table 1. During the study period, 6,080 participants developed T2D. Additional file 1: Table S1 depicts the unadjusted HRs for the incidence of T2D. Figure 2 presents the adjusted HRs of lipid profiles for the incidence of T2D. Multivariable analysis demonstrated that LDL-C (per 10 mg/dl, HR: 1.02 [95% CI 1.02–1.03]), HDL-C (per 10 mg/dl, HR: 0.88 [95% CI 0.86–0.90]), TG (per 10 mg/dl, HR: 1.008 [95% CI 1.006–1.010]), and the ratio of TG/HDL-C (HR: 1.03 [95% CI 1.02–1.03]) were linked to an increased risk of developing T2D. The multivariable analysis showed the c-index of 0.88.

**Table 1:** Characteristics of participants at baseline

N	120,613
Age (y)	44.2 (8.5)
Male, n, (%)	91,659 (76.0)
Body mass index (kg/m^2^)	22.9 (3.4)
Systolic blood pressure (mmHg)	118.7 (14.8)
Diastolic blood pressure (mmHg)	74.0 (11.1)
Low-density lipoprotein cholesterol (mg/dL)	123.4 (31.5)
High-density lipoprotein cholesterol (mg/dL)	60.5 (15.4)
Triglycerides (mg/dL)	110.0 (85.9)
Fasting plasma glucose (mg/dl)	92.6 (9.3)
Smoking (none/past/current), n, (%)	63,050/16,475/41,088 (52.3/13.7/34.1)
Physical exercise, n, (%)	20,433 (16.9)

Data are presented as mean (standard deviation) or absolute number (percentage)

The likelihood ratio test showed that lipid profiles, including TG/HDL cholesterol ratio, had a significant prognostic impact (p < 0.001) (Table 2).

**Table 2:** Examination of the model (seven variables to be forced in + additional lipid profiles)

Variables added	-2LL_0	-2LL_1	AIC	-2(LL_0-LL_1)	df	p-value
LDL cholesterol	122,330.5	122,296.4	122,314.4	34.06	1	< 0.001
HDL cholesterol		122,179.5	122,197.5	150.98	1	< 0.001
TG		122,264.3	122,282.3	66.16	1	< 0.001
TG/HDL cholesterol ratio		122,265.5	122,283.5	64.96	1	< 0.001

Seven variables; age, sex, body mass index, systolic blood pressure, fasting plasma glucose, smoking habits, and physical exercise

LDL, low-density lipoprotein; HDL, high-density lipoprotein; TG, Triglycerides

-2LL_0; The -2 log likelihood of the model with the 7 variables to be forced in as main effects

-2LL_1; The -2 log likelihood of the model with the above 7 variables + additional lipid profiles

-2(LL_0-LL_1); Test statistic

df; Degrees of freedom of the test statistic (difference in the number of parameters)

Table 3 displays the AUC and optimal cut-off values after ten years of time-dependent ROC curve analysis. The AUC and optimal cut-off values for LDL-C, HDL-C, TG, and the ratio of TG/HDL-C for incident T2D were 0.609 and 124 mg/dL, 0.638 and 54 mg/dL, 0.672 and 106 mg/dl, and 0.679 and 2.1, respectively, at 10 years. Table 3 also compares the AUCs of LDL-C, HDL-C, TG, and the ratio of TG/HDL-C. The AUC of the ratio of TG/HDL-C was greater than that of LDL-C (difference value, 0.069; 95% CI 0.061–0.078; P < 0.001), HDL-C (difference value, 0.041, 95% CI 0.035–0.046, P < 0.001), or TG (difference value, 0.007; 95% CI 0.004–0.009; P < 0.001).

**Table 3:** The comparison of area under the curve of LDL cholesterol, HDL cholesterol, triglycerides, and triglycerides/HDL cholesterol ratio

	vs. LDL cholesterol			vs. HDL cholesterol			vs. Triglycerides		
	Difference value	95% CI	P	Difference value	95% CI	P	Difference value	95% CI	P
LDL cholesterol AUC (95% CI); 0.609 (0.602–0.617) Cut-off value; 124 mg/dl	Reference			–			–		
HDL cholesterol AUC (95% CI); 0.638 (0.631–0.645) Cut-off value; 54 mg/dl	0.029	0.019–0.038	< 0.001	Reference			–		
Triglycerides AUC (95% CI); 0.672 (0.665–0.678) Cut-off value; 106 mg/dl	0.063	0.053–0.072	< 0.001	0.034	0.027–0.041	< 0.001	reference		
Triglycerides /HDL cholesterol ratio AUC (95% CI); 0.679 (0.672–0.684) Cut-off value; 2.1	0.069	0.061–0.078	< 0.001	0.041	0.035–0.046	< 0.001	0.007	0.004–0.009	< 0.001

LDL, low-density lipoprotein; AUC, area under the curve; HDL, high-density lipoprotein

### Subgroup analyses

The adjusted HRs of lipid profiles for the occurrence of T2D in subgroup analysis according to sex and BMI category are shown in Fig. 2. Additional file 2: Table S2 shows the AUC and cut-off values according to sex and BMI category. We discovered an interaction effect between sex and LDL-C and TG. The HR of LDL-C (p for interaction < 0.001) and TG (p for interaction = 0.002) was higher in females than in males. In males, LDL-C (HR: 1.02 [95% CI 1.01–1.03]) and TG (HR: 1.008 [95% CI 1.006–1.009]) were substantially related to the probability of developing T2D. In females, LDL-C (HR: 1.09 [95% CI 1.06–1.13]) and TG (HR: 1.02 [95% CI 1.01–1.03]) were substantially linked with the probability of developing T2D. The optimal ratio of TG/HDL-C cut-off values for T2D development at 10 years were 2.1 and 1.2 in males and females, respectively.

Furthermore, we discovered an interaction impact between the BMI category and TG, as well as the ratio of TG/HDL-C. The HR of TG (p for interaction = 0.0001) and TG/HDL-C (p for interaction = 0.0001) was higher in participants with a BMI of < 25 kg/m^2^ than in those with a BMI of ≥ 25 kg/m^2^. In participants with a BMI < 25 kg/m^2^, TG (HR: 1.01 [95% CI 1.010–1.014]) and the ratio of TG/HDL-C (HR: 1.04 [95% CI 1.03–1.05]) were substantially linked with the development of T2D. In participants with a BMI ≥ 25 kg/m^2^, TG (HR: 1.006 [95% CI 1.003–1.008]) and the ratio of TG/HDL-C (HR: 1.02 [95% CI 1.02–1.03]) were substantially linked with the probability of developing T2D. The optimized cut-off values for the ratio of TG/HDL-C for T2D development at 10 years were 1.7 and 2.5 in participants with BMI < 25 kg/m^2^ and BMI ≥ 25 kg/m^2^, respectively.

## Discussion

The following are the study's three key findings. First, the ratio of TG/HDL-C cut-off value for T2D development within 10 years was 2.1. Second, the ratio of TG/HDL-C outperformed LDL-C, HDL-C, and TG levels in predicting the development of diabetes within 10 years. Third, females and those with a BMI of < 25 kg/m^2^ may be more sensitive to lipid profile levels in terms of the risk of developing T2D.

Though the precise mechanism through which a high ratio of TG/HDL-C causes insulin resistance is unknown, several reports suggest a theory. In cellular experiments, LDL-C was shown to decrease the expression of cyclin B1 in pancreatic β-cells, resulting in increased insulin resistance, and HDL-C is thought to improve insulin resistance by suppressing the effects of LDL-C [20,21]. HDL has also been shown to possibly regulate glucose homeostasis through mechanisms such as insulin secretion, direct glucose uptake by muscle, and increased insulin sensitivity [22]. In contrast, hypertriglyceridemia increases free fatty acids, which accumulate in skeletal muscle and cause insulin resistance, while accumulation in pancreatic islets has been reported to cause β-cell dysfunction and apoptosis [23]. Moreover, hypertriglyceridemia increases the activity of cholesteryl ester transfer protein (CETP) [24,25]. While CETP enhances TG enrichment of HDL and LDL by causing cholesteryl ester to be converted to TG, it leads to lower HDL-C concentrations [24,25]. These findings may be related to the fact that the ratio of TG/HDL-C was more beneficial than LDL-C, HDL-C, and TG levels in predicting diabetes development. In addition, insulin resistance increases TG levels and reduces HDL-C through compensatory hyperinsulinemia and activation of fatty acid degradation [26], and in this regard, the ratio of TG/HDL-C is an important finding in conducting diabetes care.

Subgroup analysis revealed that an elevated lipid profile is more likely to affect diabetes development in females than in males and in those with a BMI of < 25 kg/m^2^ than in those with a BMI of ≥ 25 kg/m^2^. Generally, females have lower LDL-C and higher HDL-C levels than males. Due to the effect of female hormones, females have lower LDL-C levels and higher HDL-C levels than males of the same age [27]. Even though estrogen is known to increase TG levels [28], females still have lower TG levels than males. Females have been reported to have greater TG uptake by muscle cells and higher TG clearance than males [29]. Given that estrogen deficiency causes dysregulation of lipid metabolism and accumulation of visceral adipose tissue [30], it is possible that estrogen suppresses TG elevation indirectly by inhibiting the accumulation of visceral adipose tissue. In addition, females are known to be more sensitive to TG levels with respect to cardiovascular disease risk than males [31], suggesting that these results might be consistent with our findings. Furthermore, BMI is strongly connected to the risk of acquiring T2D [32], and several studies have linked increased visceral fat to insulin resistance [33]. Compared to participants with low BMI, the effect of BMI on insulin resistance may have been greater than the effect of lipid profiles in participants with high BMI. Females and individuals with a BMI < 25 kg/m^2^ may be more sensitive to levels of lipid profiles based on these findings.

Previous studies have evaluated the association between categorical or continuous TG/HDL-C ratio values and the incidence of T2D [13–18]. Kim et al. [13] and Lim et al. [14] conducted a study in a Korean population, Liu et al. [15] conducted a study in Chinese individuals (aged 75 years and older), Tohidi et al. [16] have studied the relationship in Iranians, and Wang et al. [17] investigated the relationship in Japanese people. While these studies have discovered a significant association between higher ratios of TG/HDL-C and an increased risk of new-onset diabetes, cut-off values and comparisons among lipid profiles were not presented [13–17], unlike in our study, which estimated cut-off values and compared ratio of TG/HDL-C, LDL-C, HDL-C, and TG parameters. Additionally, the sample size of some studies was not large enough [14–16]. Although Hadaegh et al. [18] reported the cut-off values of the ratio of TG/HDL-C, their sample size was small and they did not compare the performance among lipid profiles for predicting T2D incidence. Furthermore, the cut-off values of the TG/HDL-C ratio reported in their study were higher than the values reported by us [18]. This may be because their study was conducted in a Middle Eastern population, in which the pathophysiology of diabetes may be different from that in Japanese people.

It is also noteworthy that our study's sample size was large, and the follow-up period was lengthy, due to the use of a large corporate health checkup database. Furthermore, several dyslipidemia medications, such as statins, are known to increase the likelihood of developing diabetes [34,35], and by excluding participants taking these drugs, we avoided this confounding effect on our risk assessment.

This study had several limitations. First, only reasonably young Japanese were included. It is unknown whether our findings apply to other age and ethnic groups. Most of the participants in our study were males. Therefore, further research will be needed on sex differences in the relationship between lipid profiles and the development of T2D. Additionally, our study did not take into account other factors, such as genetic predisposition, diet, and medication other than antidiabetic drugs, that might influence diabetes risk. Furthermore, neither HbA1c nor a 75-g oral glucose tolerance test was employed in the diagnosis of T2D. The data of a self-reported history of diabetes or usage of antidiabetic medicines were collected using the self-administered questionnaire. Therefore, some participants with T2D might not have been identified. Finally, the lipid data was only available at baseline and was not tracked over time.

## Conclusions

In conclusion, it was demonstrated that the ratio of TG/HDL-C was a stronger predictor of T2D development within 10 years than LDL-C, HDL-C, or TG. These results indicated the importance of providing future medical care support.

### Supplementary Information

**Additional file 1: Table 1.** Unadjusted hazard ratios for incidence of diabetes during the 10-year follow-up period.

**Additional file 2: Table 2.** The area under the curve and optimal cut-off values according to sex and BMI category.

## Data Availability

The data that support the findings of this study are available from the corresponding author upon reasonable request.

## Abbreviations

AUC: Area under the curve
BMI: Body mass index
CI: Confidence interval
HDL-C: High-density lipoprotein cholesterol
HR: Hazard ratio
LDL-C: Low-density lipoprotein cholesterol
ROC: Receiver operating characteristic
T2D: Type 2 diabetes
TG: Triglyceride
