# Supplementary material for: Triglycerides/HDL cholesterol ratio and type 2 diabetes incidence: Panasonic Cohort Study 10
Source: Cardiovasc Diabetol. 2023 Nov 8;22:308. doi: 10.1186/s12933-023-02046-5 (PMC10634002; doi:10.1186/s12933-023-02046-5)
Supplement: Supplementary file 1 — Additional file 1: Table 1. Unadjusted hazard ratios for incidence of diabetes during the 10-year follow-up period. [file 12933_2023_2046_MOESM1_ESM.docx]

Additional Table 1. Unadjusted hazard ratios for incidence of diabetes during the 10-year follow-up period

|  | HR (95% CI) |
| --- | --- |
| Age (per 10years) | 2.04 (1.97-2.12) |
| Sex (ref: female) | 3.17 (2.88-3.49) |
| Body mass index (per 1kg/m^2^) | 1.20 (1.19-1.20) |
| Systolic blood pressure (per 10mmHg) | 1.39 (1.37-1.41) |
| Low-density lipoprotein cholesterol (per 10mg/dl) | 1.11 (1.11-1.12) |
| High-density lipoprotein cholesterol (per 10mg/dl) | 0.70 (0.69-0.72) |
| Triglycerides (per 10mg/dl) | 1.022 (1.021-1.023) |
| Triglycerides/High-density lipoprotein cholesterol ratio | 1.07 (1.06-1.07) |
| Fasting plasma glucose (per 10mg/dl) | 4.29 (4.18-4.39) |
| Smoking (past) (ref: none) | 1.38 (1.28-1.49) |
| Smoking (current) (ref: none) | 1.73 (1.64-1.82) |
| Physical exercise (yes) (ref: no) | 1.10 (1.03-1.17) |
