# Supplementary material for: Triglycerides/HDL cholesterol ratio and type 2 diabetes incidence: Panasonic Cohort Study 10
Source: Cardiovasc Diabetol. 2023 Nov 8;22:308. doi: 10.1186/s12933-023-02046-5 (PMC10634002; doi:10.1186/s12933-023-02046-5)
Supplement: Supplementary file 2 — Additional file 2: Table 2. The area under the curve and optimal cut-off values according to sex and BMI category. [file 12933_2023_2046_MOESM2_ESM.docx]

Additional Table 2. The area under the curve and optimal cut-off values according to sex and BMI category

| Male | AUC | Sensitivity | Specificity | Cut-off value |
| --- | --- | --- | --- | --- |
| Low-density lipoprotein cholesterol | 0.587 | 62.9 % | 50.6 % | 124 mg/dl |
| High-density lipoprotein cholesterol | 0.612 | 56.4 % | 61.2 % | 53 mg/dl |
| Triglycerides | 0.647 | 65.1 % | 57.4 % | 106 mg/dl |
| Triglycerides /HDL cholesterol ratio | 0.653 | 62.3 % | 61.5 % | 2.1 |
| Female | AUC | Sensitivity | Specificity | Cut-off value |
| Low-density lipoprotein cholesterol | 0.713 | 67.0 % | 65.6 % | 122 mg/dl |
| High-density lipoprotein cholesterol | 0.692 | 56.4 % | 76.3 % | 60 mg/dl |
| Triglycerides | 0.744 | 73.6 % | 63.3 % | 69 mg/dl |
| Triglycerides /HDL cholesterol ratio | 0.756 | 67.5 % | 72.5 % | 1.2 |
| BMI < 25kg/m^2^ | AUC | Sensitivity | Specificity | Cut-off value |
| Low-density lipoprotein cholesterol | 0.592 | 55.5 % | 59.3 % | 124 mg/dl |
| High-density lipoprotein cholesterol | 0.598 | 48.6 % | 66.4 % | 56 mg/dl |
| Triglycerides | 0.650 | 54.3 % | 68.8 % | 101 mg/dl |
| Triglycerides /HDL cholesterol ratio | 0.650 | 55.0 % | 67.4 % | 1.7 |
| BMI ≥ 25kg/m^2^ | AUC | Sensitivity | Specificity | Cut-off value |
| Low-density lipoprotein cholesterol | 0.550 | 61.2 % | 46.8 % | 130 mg/dl |
| High-density lipoprotein cholesterol | 0.576 | 63.2 % | 49.7 % | 52 mg/dl |
| Triglycerides | 0.589 | 71.1 % | 42.4 % | 108 mg/dl |
| Triglycerides /HDL cholesterol ratio | 0.597 | 61.0 % | 54.1 % | 2.5 |

Abbreviations: HDL, high-density lipoprotein; AUC, area under the curve
